# Development of a four-gene host signature for paucibacillary TB among symptomatic individuals with sputum Xpert MTB/RIF Ultra very low and trace results

**DOI:** 10.1016/j.ijid.2026.108763

**Published:** 2026-05-03

**Authors:** Kattya Lopez, Robert Reiss, Vaishnavi Kaipilyawar, Arthur VanValkenburg, Xutao Wang, Nisha Modi, Emily Hennig, Aanchal Wats, Christie Eichberg, Lydia Nakiyingi, Chad Centner, Kimberly McCarthy, Jerrold J. Ellner, David Alland, Susan E. Dorman, W. Evan Johnson, Yingda L. Xie, Padmini Salgame

**Affiliations:** 1 Department of Medicine and Public Health Research Institute, Rutgers New Jersey Medical School, Newark, NJ, United States; 2 Department of Medicine, Medical University of South Carolina, Charleston, SC, United States; 3 Infectious Diseases Institute, Makerere University, Kampala, Uganda; 4 Division of Medical Microbiology, University of Cape Town Faculty of Health Sciences, Cape Town, South Africa; 5 US Centers for Disease Control and Prevention, Kisumu, Kenya

**Keywords:** Biomarker, Tuberculosis, Xpert MTB/RIF Ultra

## Abstract

**Objectives:**

The Xpert MTB/RIF Ultra (Ultra) assay is widely used to diagnose pulmonary tuberculosis (TB), but “very low” or “trace” results may reflect either paucibacillary TB or TB-negative disease, complicating clinical decision-making. We evaluated host transcriptomic signatures to identify culture-confirmed paucibacillary TB among individuals with Ultra very low or trace results.

**Methods:**

We performed whole blood targeted transcriptional profiling of 90 symptomatic adults from Uganda, Kenya, and South Africa with Ultra very low/trace sputum results. An 81-gene customized NanoString panel representing 13 published TB signatures was analyzed using machine learning to derive a novel four-gene host signature (“TRACE4”) predicting MGIT and LJ culture positivity. Validation included individuals with other respiratory diseases (n = 18) and North American-TB-negative controls (n = 20). Data were randomly split 75/25 into training (n = 67) and test (n = 23) sets. Diagnostic performance was evaluated against WHO targets.

**Results:**

TRACE4 outperformed all published signatures in both training (AUC 0.89) and test sets (AUC 0.88), achieving 82% specificity at 75% sensitivity. It also exceeded reclassification based on prior TB history (specificity 0.58 (95% CI: 0.45–0.69); sensitivity 0.35 (95% CI: 0.15–0.61)). TRACE4 showed 100% specificity in non-TB controls.

**Conclusion:**

TRACE4 shows promise for identifying paucibacillary culture-positive pulmonary TB in this diagnostically challenging group.

## Introduction

Tuberculosis disease (TB) is one of the deadliest diseases worldwide [1], with 10.8 million active cases and 1.25 million deaths reported in 2023. Of these, 6.1% were in people with HIV and 24% in Africa, highlighting the region’s burden. The COVID-19 pandemic further disrupted TB detection and treatment. To achieve the goal of ending the Global TB Epidemic by 2035, better diagnostic tools are urgently needed.

Xpert MTB/RIF Ultra (“Ultra”, Cepheid) is a WHO-recommended rapid molecular test that detects pulmonary TB and rifampicin resistance [2]. It offers improved sensitivity compared with earlier molecular assays, particularly in paucibacillary TB, and large multicenter studies have demonstrated that Ultra sensitivity is non-inferior to that of culture. Ultra results are categorized semiquantitatively (high, medium, low, very low, trace), with “trace” results comprising 3–30% of Ultra-positives cases [3]. However, the increased sensitivity of Ultra has been shown to come at the expense of reduced specificity, particularly in paucibacillary TB disease [4]. Accordingly, “very low” and “trace” results are associated with limited positive predictive value (33–50%) for culture-confirmed pulmonary TB, often leading to diagnostic uncertainty [5–7]. The WHO therefore recommends interpreting “very low” and “trace” results cautiously and incorporating clinical context, including prior TB history, HIV status, and abnormal chest X-rays into treatment decisions [2].

Ambiguity surrounding “trace” and “very low” results poses major clinical challenges, especially in high-burden settings where false positive results can lead to overtreatment and false negative results may delay care. These limitations highlight the need for complementary, rapid and accessible tools to determine if such results represent true paucibacillary TB. We reasoned that a host blood transcriptional signature could distinguish active TB disease from non-viable *Mycobacterium tuberculosis* (Mtb) DNA or contamination, which would not be expected to elicit a TB-specific host response. In this study conducted in Kenya, South Africa and Uganda, we evaluated the performance of existing TB gene signatures and developed a novel parsimonious 4-gene signature (TRACE4) to identify culture-confirmed paucibacillary TB among symptomatic individuals whose sputum Ultra result was “very low” or “trace.”

## Methods

### Human participants

Participants were recruited from a prospective cohort of symptomatic adults undergoing evaluation for TB consecutively enrolled in Kenya, South Africa and Uganda between July 2018 and May 2019 [7]. Inclusion criteria were age ≥ 18 years, symptoms associated with pulmonary TB (>2 weeks of cough and at least one additional tuberculosis sign/symptom (e.g., fever, night sweats, weight loss or hemoptysis), and had not received anti-TB treatment within 6 months before enrollment. Three sputum samples were collected over 2 days. Smear microscopy and Ultra were performed from Sputum 1. Sputum 2 and 3 were used for culture on Löwenstein–Jensen (LJ) and Mycobacteria Growth Indicator Tube (MGIT) media. Clinical evaluation and microbiological procedures are detailed in Xie *et al.* [7].

This sub-study included only participants whose highest Ultra-positive result was “very low” or “trace” and MGIT or LJ culture results were available from baseline visits. Adults from North America, who were unlikely to have been exposed to Mtb and had negative QuantiFERON test results within the past 2 years, were enrolled as healthy “negative controls” for this sub-study. 18 participants with non-TB other respiratory diseases such as COPD, viral respiratory infections, bronchopneumonia, and pulmonary edema recruited at University Hospital, Newark, NJ, affiliated with Rutgers (NJ-ORD) and confirmed to be sputum Mtb PCR-negative were also evaluated. All adult study participants provided informed consent approved by ethics committees at each study site institution.

### Samples

Whole blood samples from baseline visits, US-based negative controls and NJ-ORD participants were collected directly into PAXgene blood RNA tubes (Cat. no. 762165, Qiagen, Hilden, Germany) following the manufacturer’s recommendations and stored at −80°C until processing. RNA was subsequently isolated and purified using the PAXgene Blood RNA kit (Cat. no. 762174, Qiagen, Hilden, Germany) according to the kit handbook. RNA was quantified and purity was determined using a NanoDrop One spectrophotometer.

### Analysis of host response signatures using machine learning and profiling tools

To examine TB biomarkers, gene expression analysis was conducted using the NanoString platform following the manufacturer’s protocol. A customized NanoString TB panel of 81 genes (NS-TB81) was utilized, comprising 13 published TB signatures and six housekeeping genes. These transcriptomic signatures differentiate between TB disease and healthy controls, latent TB and HIV-coinfection, Maertzdorf4 [8] Sweeney-OD3 [9], Jacobsen3, Kulkarni-HIV2, Roe3, Sambarey-HIV10, Sloot-HIV2 and Zhao-NANO6 (referenced in [10]). The panel also included signatures associated with progression to TB disease, including Darboe-RISK11 [11], PREDICT29 [12], RISK6 [13], Suliman-RISK4 [14] and ZAK-RISK16 [15], as well as an early treatment-response signature [16]. The data were analyzed with nSolver 4.0 Analysis Software System and the R-based computational platform TB “TBSignatureProfiler” (TBSP) [17]. Normalized gene expression data were used to calculate signature scores for each sample. Non-parametric, unsupervised gene set variation analysis (GSVA) was applied to compare the signatures between Ultra-positive very low or trace results and Mtb culture-positive or Mtb culture-negative samples. Machine learning analysis using the AutoML function from the H2O.ai (Mountain View, CA) R package was performed on training and test datasets to classify Mtb culture status. AutoML systematically trains and optimizes multiple machine learning algorithms (including random forests, gradient boosting machines and deep learning models) under a unified framework. To ensure robustness given the relatively small sample size, model training incorporated internal bootstrap-based cross-validation, whereby the training data are repeatedly resampled to evaluate model performance and reduce overfitting.

Feature selection was based on the consistency of gene importance rankings across multiple model types, prioritizing genes that demonstrated reproducible predictive value across diverse algorithms. This approach reduces reliance on any single model and enhances the stability of the selected biomarkers. The final four-gene signature was then used to derive an enrichment-based biomarker score using the TBSignatureProfiler. Details of the four-gene signature selection are provided in the Results section.

Model performance was evaluated using boxplots, receiver operating characteristic (ROC) curves and area under the ROC curve (AUC) metrics. Sensitivity and specificity were calculated according to the minimum WHO Target Product Profile (TPP) criteria for a non-sputum near point-of-care diagnostic test [18].

### Evaluation of biomarker specificity and cross-dataset validation

To determine biomarker specificity, we also collected PAXgene blood tubes, extracted RNA, and performed gene expression analysis using NanoString technology from the two North-American cohorts: TB-negative controls and participants with other respiratory diseases but negative for TB and COVID-19. The curatedTBData provides access to TB transcriptomic studies and enables comparisons of TB gene signatures of interest across multiple published datasets [19]. We used this platform to assess the performance of the newly developed 4-gene signature on published available datasets of other respiratory diseases.

### Statistics

Processed data were analyzed using R version 4.2.3. Baseline characteristics of study participants were presented by frequency for categorical variables. Categorical variables (demographics and clinical data) were compared using Fisher’s exact test (2 × 2-table) or Pearson’s chi-square test. Using culture status as the reference, sensitivity and specificity were calculated for the TRACE4, Darboe-RISK11, Zak-RISK16 and Maertzdorf4 signatures via ROC analysis using the pROC package. Sensitivity and specificity were also calculated for prior TB history and clinical diagnosis by the seven-day follow-up visit via confusion matrix analysis. 95% confidence intervals were calculated via bootstrap resampling. *P*-values < 0.05 were considered statistically significant.

## Results

### Demographics

Among 1199 consecutively-enrolled symptomatic patients undergoing evaluation for tuberculosis (TB), 321 were Xpert MTB/RIF Ultra (Ultra) positive and had MGIT and LJ culture results, of whom 35 (15.8%) were Ultra semiquantitative value “very low” and 55 (24.8%) were Ultra “trace” (Figure 1). Among these 90 participants included in the study, 17 (18.8%) had culture-positive TB and 73 (81.1%) were culture-negative. Baseline characteristics of the study population are shown in Table S1. The proportion of females (58.8% vs 42.5%) and individuals living with HIV (64.7% vs 50.7%) was higher in the Mtb culture-positive group compared to the culture-negative group, although neither difference was statistically significant. A prior TB history was reported in 35.3% of culture-positive participants and 42.5% of culture-negative participants. All participants presented with cough, whereas the prevalence of other symptoms, including fever, night sweats, weight loss, and hemoptysis, showed no significant differences between groups. Smear microscopy was negative in 94.1% of culture-positive and 98.6% of culture-negative individuals. At the 7-day follow up, 75.0% of culture-positive participants were diagnosed with active pulmonary TB compared to 32.9% in the culture-negative group (*P* < 0.01), with four participants missing TB diagnosis data. The dataset (n = 90) was split 75/25 into training (n = 67) and test (n = 23) sets and baseline characteristics per set are shown in Tables S2 and S3.

### Performance of published gene signatures

We evaluated the performance of 13 published TB host gene signatures using the R package TBSignatureProfiler (TBSP) (Table S4). Gene Set Variation Analysis (GSVA) identified Maertzdorf4 as the top-performing signature, achieving an AUC of 0.73 (*P* = 0.005). Zak-RISK16 and Darboe-RISK11 followed closely, with AUC values of 0.70 (*P* = 0.02). However, the remaining signatures displayed lower AUC values (≤0.63) and did not achieve statistically significant performance, highlighting the variability in predictive accuracy across existing gene signatures.

### Derivation and validation of a four-gene host signature for Mtb culture prediction

#### Data preparation and partitioning:

The study dataset included predictor variables (TB history and NS-TB81 gene signature scores) and the binary outcome variable, Mtb culture status (positive or negative). The dataset was divided into training (75%, n = 67) and test (25%, n = 23) sets using stratified random sampling to ensure balanced representation of Mtb culture-positive and culture-negative participants. The test set was reserved for independent validation of the four-gene host signature. A fixed random seed was used to ensure reproducibility of the data partitioning; the exact seed value and code are provided in the GitHub repository referenced in the Data Availability Statement.

#### Application of multiple machine learning models:

The autoML machine learning framework in the H2O.ai R package was employed for model training and evaluation. The dataset was imported into the H2O platform and models, including generalized linear models (GLMs), random forests, and gradient boosting machines (GBMs), were trained to classify Mtb culture status. Hyperparameters were fine-tuned using an automated grid search to improve the performance of the predictive model.

#### Gene importance and signature derivation:

Variable importance scores, representing the contribution of individual genes to each model’s predictions, were generated by the H2O platform. The top-ranking genes across models were identified as potential candidates for further analysis. Using the TBSP platform, these top genes were used to profile new gene signatures to predict Mtb culture status among participants with Ultra “very low” or “trace” results. This process resulted in the derivation of a novel four-gene host signature comprising ZNRF1, IL31RA, SRBD1, WARS. This set of genes was named “TRACE4” (Figure S1).

The genes comprising “TRACE4” are associated with innate immune signaling and include four genes with distinct roles in immune modulation. ZNRF1 (Zinc and Ring Finger 1), is an E3 ubiquitin ligase, modulates endosomal trafficking of the epidermal growth factor receptor (EGFR) and plays a role in ubiquitination [20], IL31RA (Interleukin 31 receptor A) contributes to the proliferation and activation of myeloid cells, promoting innate immune responses [21]. SRBD1 (S1 RNA-Binding Domain 1) is found in numerous RNA-associated proteins, including polynucleotide phosphorylase (PNPase), which is targeted by pyrazinoic acid, the active component of pyrazinamide, a first-line antituberculosis drug [22]. WARS (Tryptophanyl-tRNA synthetase) acts as an endogenous ligand for Toll-like receptors 2 and 4 and is induced by interferon-*γ* (IFN-*γ*) produced by both innate immune cells and T cells [23]. Interestingly, these four genes are a subset of the previously established gene signature Leong-RISK29 (PREDICT29), which has been shown to predict the risk of progression to active TB disease in recently exposed household contacts up to 5 years before disease onset [12]. All four genes of “TRACE4” are upregulated within the PREDICT29 signature, highlighting their potential relevance in TB pathogenesis and immune response.

### Diagnostic and triage performance of TRACE4 and other TB signatures

The performance of the four-gene host signature, TRACE4, was evaluated for its ability to predict Mtb culture status among participants with Ultra “very low” or “trace” results. In the training dataset, the GSVA scores for culture-positive individuals (n = 11) were significantly higher compared to culture-negative individuals (n = 56), achieving an area under the curve (AUC) of 0.89 (Figure 2A). Similarly, in the independent test dataset, TRACE4 also demonstrated strong predictive capability, with culture-positive individuals (n = 6) showing higher GSVA scores compared to culture-negative individuals (n = 17), resulting in an AUC of 0.88 (Figure 2B).

The diagnostic performance of the TRACE4 gene signature was evaluated alongside 13 published TB signatures in both the training (n = 67) and test sets (n = 23). In both training (Table S5) and test (Table 1), we evaluated TRACE4 performance against the WHO targets (TPP) for a biomarker-based non-sputum, near point-of-care diagnostic assay for all TB [18]. At the WHO minimum target sensitivity of ≥75% in the test set, TRACE4 achieved a specificity of 0.82 (Table 1).

Although TRACE4 was not explicitly developed or evaluated as a triage test (Table S6), it achieved a sensitivity of 1.00 (95% CI: 0.33–1.00) at a specificity of 0.76 (95% CI: 0.59–0.94) in the independent test dataset, meeting the WHO TPP requirement of ≥70% specificity at triage-level sensitivity. In the training set, TRACE4 demonstrated a sensitivity of 0.91 (95% CI: 0.73–1.00) and specificity of 0.80 (95% CI:0.54–0.91) (Table S5). Among all evaluated signatures, TRACE4 was the only one to consistently approach WHO TPP benchmarks across datasets.

A few symptomatic, culture-negative individuals in our cohort exhibited elevated GSVA scores for the TRACE4 signature (Figure 2), suggesting that the signature may detect host responses of TB with low or undetectable bacterial burden. To further evaluate its specificity, we validated TRACE4 in an independent cohort of other respiratory diseases (NJ-ORD). No overlap was observed between the GSVA scores of individuals expressing the positive TRACE4 signature and participants diagnosed with ORD or US-based negative controls (Figure 3), with TRACE4 achieving a specificity with respect to culture of 1.00 (95% CI: 1.00–1.00) for ORD and 1.00 (95% CI: 1.00–1.00) for negative controls.

### Diagnostic performance of clinical variables

The WHO recommends interpreting Ultra trace results in clinical context including prior TB treatment history, probability of relapse, and other test results [2]. We evaluated the diagnostic performance of TRACE4 against two clinical reference points: (i) classification of “trace” and “very low” Ultra results as remnant Mtb DNA and thus unlikely to represent active disease in individuals with prior TB, and (ii) routine clinician-assigned TB diagnosis by the Day 7 follow-up visit. Prior TB history showed low accuracy (sensitivity: 0.17 [95% CI: 0.01–0.64], specificity: 0.54 [95% CI: 0.29–0.76]), whereas clinical diagnosis by day 7 achieved higher sensitivity (1.00 [95% CI: 0.52–1.00]) but moderate specificity (0.65 [95% CI: 0.39–0.85]) (Tables 1 and 2). Importantly, TRACE4 outperformed these clinical variables in the overall dataset (Table 1), even when applied as a triage test, although formal triage performance was not evaluated in this study (Table S7).

### Diagnostic performance across TB cohorts

To assess the generalizability of the “TRACE4” signature, we used the *curatedTBData* R package, which compiles 49 publicly available TB transcriptomic datasets. Using GSVA, “TRACE4” consistently distinguished individuals with active pulmonary TB across these published cohorts (not limited to Ultra trace/very cases) from those with latent TB infection, achieving an AUC ROC of at least 0.83 across multiple cohorts (Table S8). In comparison to a published dataset of other respiratory diseases (GSE73408), we further tested whether the signature might perform as well as it did in our cohort. TRACE4 showed fair overall performance in this context, with an AUC of 0.76 in distinguishing pulmonary TB disease from individuals with other respiratory conditions (Table S8).

## Discussion

In this study of symptomatic adults from high TB-burden African countries, we developed and internally validated a four-gene blood-based host signature, TRACE4, as a reflex test to identify individuals with culture-confirmed paucibacillary TB among those with Xpert MTB/RIF Ultra (Ultra) “very low” or “trace” semiquantitative results. Using machine learning and host transcriptomic profiling, our signature achieved discriminatory performance for culture-positive paucibacillary TB that surpassed previously published TB signatures and maintained discriminatory performance compared to reclassifying participants TB status by prior TB history. Furthermore, the TRACE4 signature identified 25% (4/16) of culture-positive individuals who remained undiagnosed by the 1-week visit, though this occurred in the training set.

Despite improvements in molecular diagnostics, early recognition of paucibacillary TB remains problematic. 4/16 (25%) of culture-positive individuals with Ultra “trace” or “very low” results were not clinically diagnosed by day 7 and initiated treatment only after culture confirmation, conversely, 23/70 (33%) of culture-negative individuals were retrospectively confirmed to have TB, highlighting persistent diagnostic uncertainity in low-burden disease. Prior community-based studies in Uganda similarly shows that trace-positive cases are often culture negative and lack definitive clinical or/and radiological findings at baseline [24], yet many are subsequently confirmed with TB on longitudinal follow-up (within 2 years) [25], consistent with the delayed diagnosis seen in our study.

This diagnostic uncertainity underscores the need for improved molecular or host-based tools. The limited performance of existing host transcriptomic signatures in this cohort is likely due to the paucibacillary nature of disease, which is associated with lower inflammatory responses and potentially more subtle transcriptional changes in peripheral blood. Previously developed signatures were derived from cohorts with high bacterial burden and more pronounced immune activation, which may limit their generalizability to individuals with very low or trace bacillary loads. In simulating a reflex test applied to Ultra “very low” or “trace” participant samples, TRACE4 achieved a specificity of 0.82 at the WHO-recommended minimum sensitivity threshold of 0.75 for a near point-of-care non-sputum test. However, the associated confidence intervals were wide, reflecting uncertainty due to the limited sample size. Although TRACE4 did not meet the WHO TPP criteria of ≥75% sensitivity and >95% specificity, it is important to note that our study population was predominantly paucibacillary, representing a substantially more diagnostically challenging cohort than the general TB populations for which the WHO benchmarks were developed. The WHO TPPs are intended to apply broadly across TB disease regardless of bacterial burden, whereas TRACE4 was evaluated specifically in individuals with Ultra very low or trace results, where reduced performance is expected. Additionally, although culture was used as the reference standard, it is uncertain to what extent TRACE4 may detect culture-negative TB, in which case its specificity for TB disease would be underestimated in this study. We hypothesize that TRACE4 may identify individuals with incipient or culture-negative TB; however, this interpretation remains speculative in the absence of longitudinal follow-up data. Supporting this hypothesis, an independent longitudinal study documented progression to culture-positive TB at 2 months in one of three individuals who were TRACE4-positive, culture-negative at baseline [26].

Notably, within the full cohort, TRACE4 identified 4 of 16 culture-positive individuals who were not diagnosed at day 7 clinical follow-up, highlighting its potential to flag early or underrecognized cases that might otherwise be missed in routine care. Consistent with this, TRACE4 outperformed clinical variables such as prior TB history, which failed to reliably reclassify culture-positive and culture-negative Ultra very low/trace cases. Integrating clinical variables into the model did not enhance predictive performance, reinforcing the value of blood-based transcriptomics as a standalone tool in guiding treatment decisions.

The ability to detect paucibacillary TB is particularly relevant in high-burden, high TB-HIV prevalence settings, such as Uganda, Kenya, and South Africa, where the morbidity and mortality associated with missing a TB diagnosis often outweigh the risks associated with unnecessary treatment. In such contexts, sensitivity is often prioritized, and a test like TRACE4 can increase prompt diagnosis in individuals with true TB disease. For TRACE4 to function effectively as a diagnostic tool, it must be deliverable in a rapid, near-patient format. This could be achieved using a platform like existing molecular system, such as the Cepheid GeneXpert, which has already implemented an automated, integrated 3-gene host response fingerstick blood assay with a turnaround time ranging from 1 to 6 hours [27]. A platform capable of producing same-day results would facilitate timely clinical decision-making, particularly in resource-limited settings where delays in diagnosis can have serious consequences. In this scenario, TRACE4 implemented as a rapid cartridge-based assay could help clinicians stratify patients with paucibacillary TB at near point-of-care, thereby appropriately initiating anti-TB treatment only for those with TB disease and preventing unnecessary treatment for others. Conversely, in low-burden, low-HIV settings with robust close patient-follow up, the cutoff for TRACE4 could be adjusted to prioritize higher specificity to minimize overtreatment in populations where TB is less common and alternative diagnoses are more likely.

The genes comprising TRACE4, ZNRF1, IL31RA, SRBD1, and WARS, are part of immune response pathways and are also included in the validated PREDICT29 progression signature [12]. Their upregulation supports the idea that this signature may capture both current active disease and immunologic signals related to risk of progression, further highlighting its clinical relevance.

Although our study of pre-selected Ultra-positive individuals was not designed to evaluate TRACE4 as a triage test, we noted 76% specificity at 90% sensitivity which met the WHO’s minimum TPP for a TB triage test of >90% sensitivity and ≥70% specificity [18]. This supports future exploration of TRACE4 as a triage test for populations with a high prevalence of paucibacillary TB, but such an evaluation would require consecutive, untested populations. Additionally, the utility of a triage test for paucibacillary TB necessitates a reliable and practical confirmatory test which is currently lacking.

Interestingly, a subset of culture-negative individuals (18%, 13/73) also had elevated TRACE4 scores. Whereas this could reflect a lack of specificity, we observed no overlap between these participants and individuals with other respiratory diseases in an independent control cohort. One possibility is that the signature may be detecting host responses indicative of culture-negative TB rather than non-specific inflammation. These cases may represent Ultra-positive individuals with low-level or intermittent bacterial shedding, consistent with findings from Sung *et al.* [24], where a portion of trace-positive individuals exhibited clinical or radiologic signs of TB despite negative culture results. Recently published data from our group indicate that some culture-negative individuals may, in fact, represent true TB cases that are not captured by conventional microbiologic testing [26].

Limitations of this study include the relatively small sample size and geographic restriction to three African countries to represent Ultra very/trace individuals and to New Jersey for TB-negative controls, which may limit generalizability. The limited sample size is reflected in the wide confidence intervals observed in the test set performance estimates, indicating uncertainty around point estimates and warranting cautious interpretation. Additionally, we were unable to assess performance stratified by Ultra category (trace vs very low) or HIV status due to small and imbalanced subgroup sizes. Therefore, prospective validation in external, larger and more diverse cohorts, including individuals with HIV coinfection, prior TB, or other comorbidities, is warranted. Finally, we used sputum Mtb culture as a reference standard for paucibacillary TB, but it is possible that a subset of culture-negative individuals may represent culture-negative TB [26]. This translates into a possible underestimation of specificity for paucibacillary TB. Nonetheless, TRACE4 represents a promising biomarker to guide diagnosis and treatment decisions in individuals with challenging Ultra results.

## Conclusion

In conclusion, TRACE4 is a promising reflex test to identify culture-confirmed paucibacillary TB among individuals with Ultra very low or trace results. With further validation and adaptation for near point-of-care use, this signature has the potential to reduce diagnostic gaps in the early detection of paucibacillary TB and support efforts to reduce missed cases and accelerate treatment initiation globally.

## Supplementary Material

Supplementary material

Supplementary material associated with this article can be found, in the online version, at doi:10.1016/j.ijid.2026.108763.

## Figures and Tables

**Figure 1.**
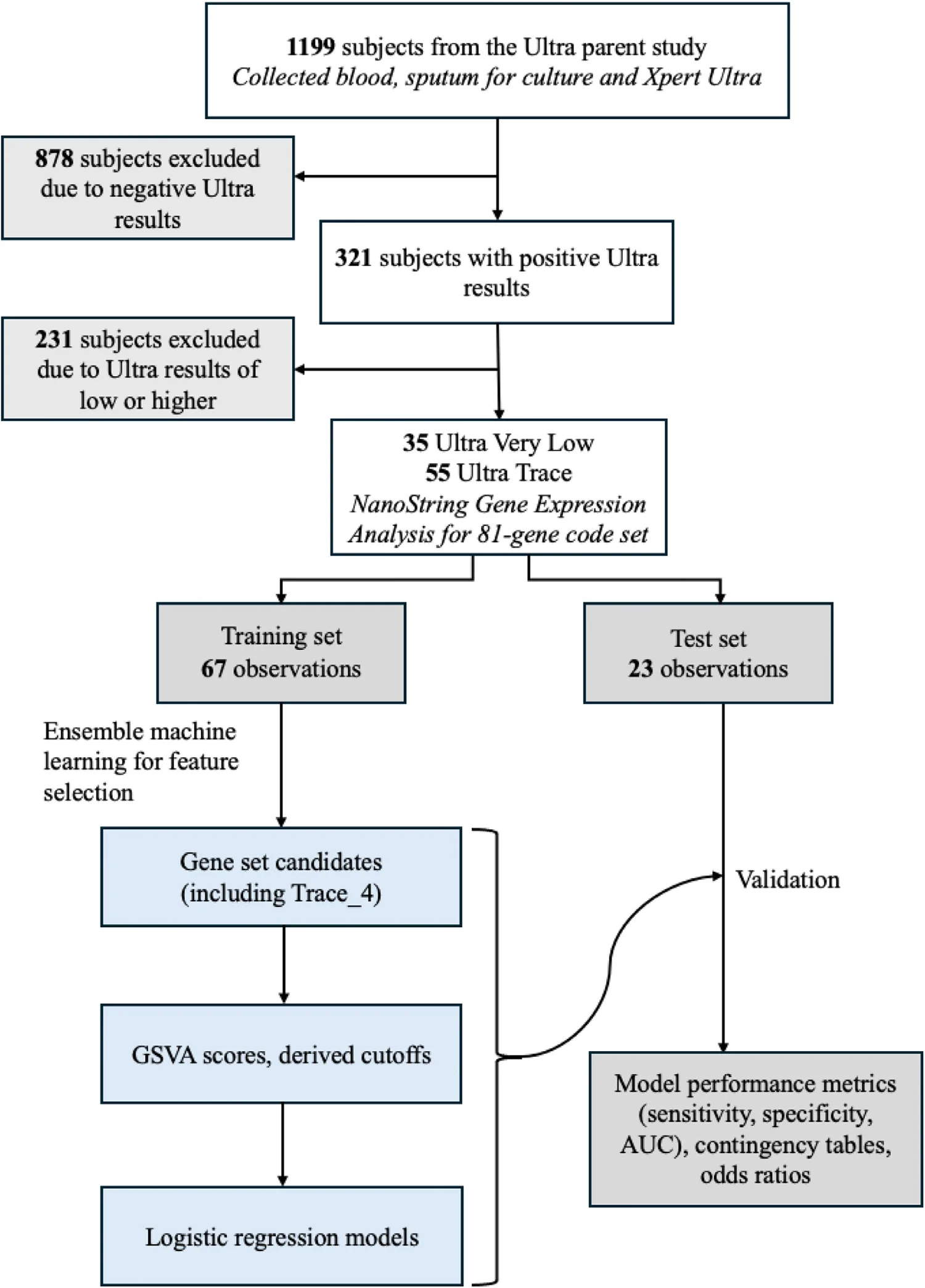
Flow diagram for study participants, machine learning and statistical analysis.

**Figure 2.**
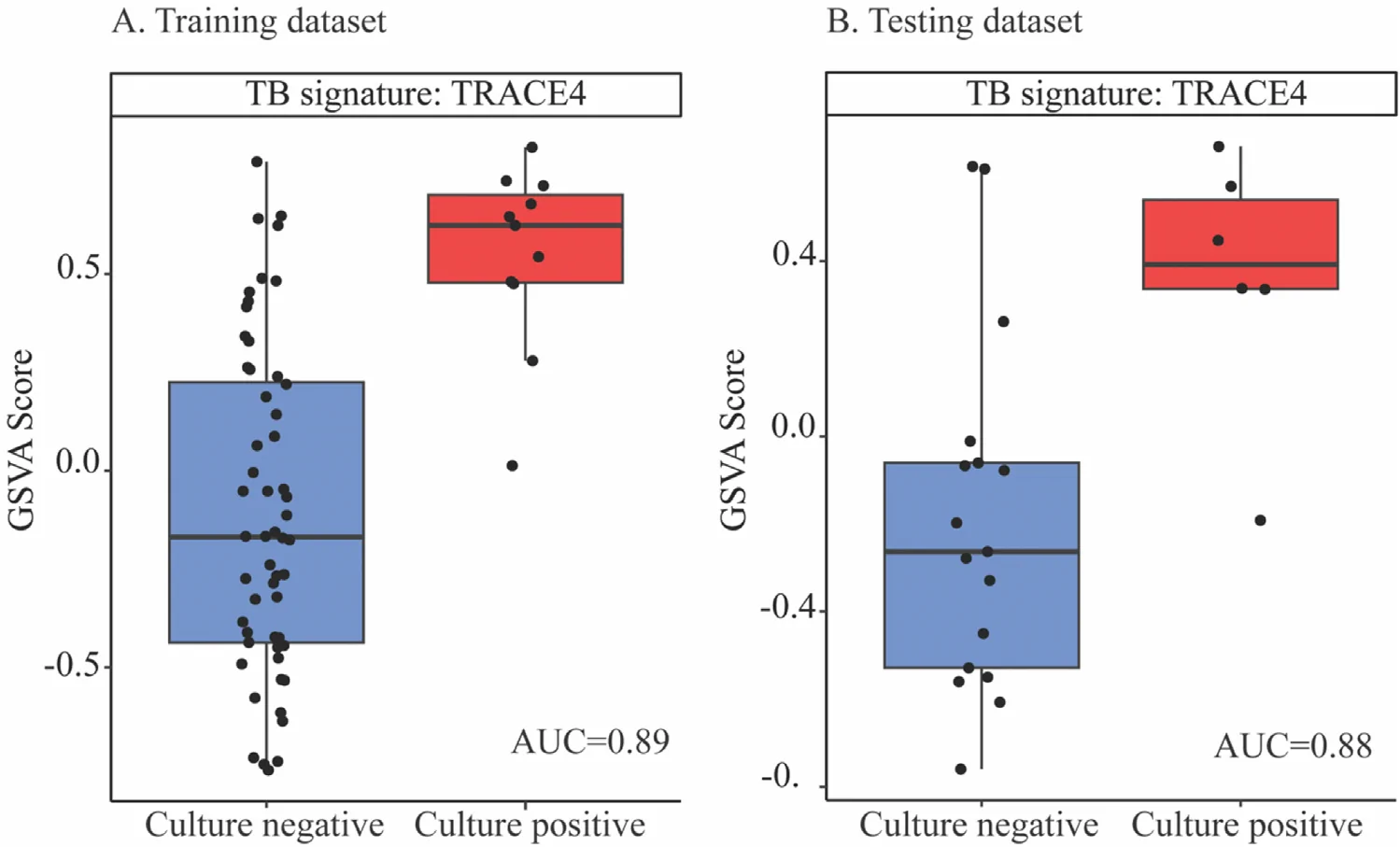
Performance of TRACE4 to predict Mtb culture status among individuals with Ultra very low or trace results. A). Training dataset, culture negative (n = 56) and culture positive (n = 11). B). Testing dataset, culture negative (n = 17), culture positive (n = 6). Abbreviations: AUC, area under the curve; GSVA, gene set variation analysis; TB, tuberculosis.

**Figure 3.**
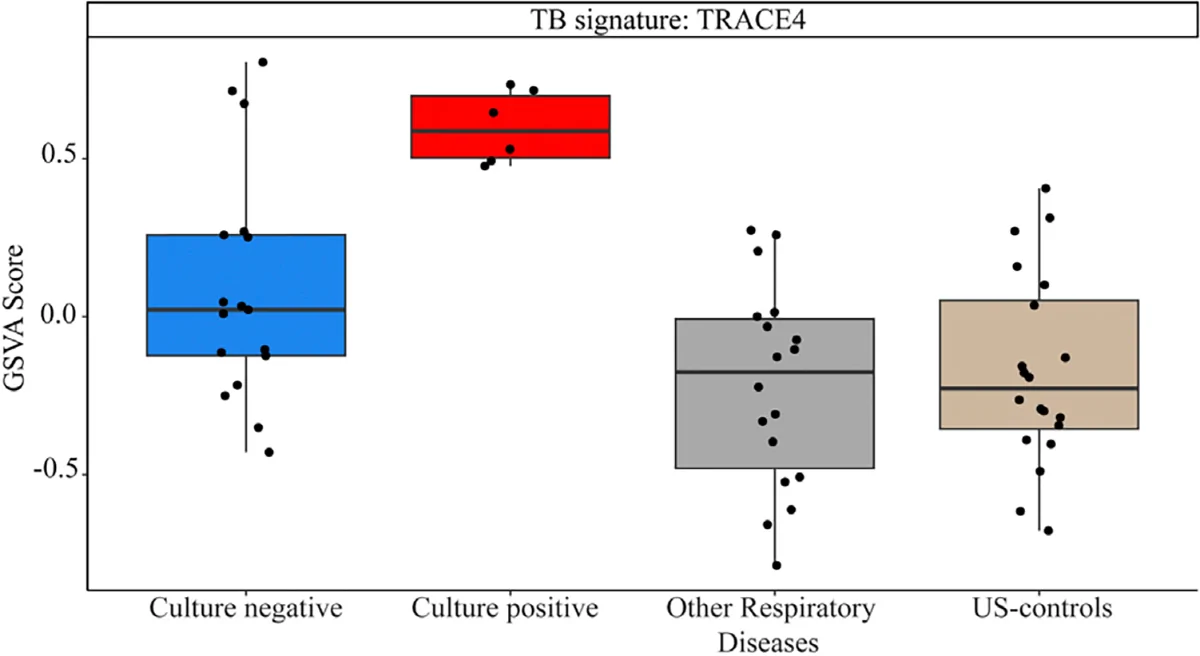
Performance of TRACE4 signature among individuals with other respiratory diseases (n = 18; pneumonia, chronic obstructive pulmonary disease, asthma, Kaposi’s sarcoma, granulomatosis with polyangiitis, *Mycobacterium avium* lung disease) and individuals without signs of exposure to tuberculosis (US-controls, n = 20). Testing dataset; culture negative (n = 17) and culture positive (n = 6). Abbreviations: GSVA, gene set variation analysis; TB, tuberculosis; US, United States.

**Table 1:** Performance metrics of prior TB history, clinical TB diagnosis by day 7, and the top four transcriptomic signatures for classifying culture status among Ultra “very low” or “trace” Xpert-positive participants (test set n = 23, full dataset n = 90).

	Specificity, Test Set [95% CI]	Sensitivity, Test Set [95% CI]	Specificity, Full Dataset [95% CI]	Sensitivity, Full Dataset [95% CI]

Prior TB history^b^	0.53 [0.29, 0.76]	0.17 [0.01, 0.64]	0.58 [0.45, 0.69]	0.35 [0.15, 0.61]
Clinical TB diagnosis by day 7	0.65 [0.39, 0.85]	1.00 [0.52, 1.00]	0.66 [0.53, 0.76]	0.75 [0.47, 0.92]

	Specificity^a^, Test Set [95% CI]^a^	Sensitivity, Test Set (Fixed)	Host Response Signature cutoff	AUC [95% CI]

**TRACE4**	0.82 [0.59, 1.00]	0.75	0.290	0.88 [0.71, 0.99]
Darboe-RISK11	0.65 [0.12, 1.00]	0.75	−0.048	0.76 [0.51, 0.96]
Zak-RISK16	0.46 [0.14, 0.79]	0.75	0.001	0.74 [0.49, 0.93]
Maertzdorf4	0.47 [0.24, 0.88]	0.75	−0.269	0.70 [0.47, 0.90]

a Specificity is calculated at 0.75 sensitivity, the WHO TPP required minimum for a biomarker-based non-sputum-based near point-of-care diagnostic assay for detecting TB.

b Prior TB history: consideration of a trace Ultra result as likely remnant Mtb DNA from prior TB, and thus TB-negative status. One individual is missing results for prior TB history in the test set, and four individuals are missing results in the full dataset.Diagnostic performance was assessed based on association with culture positivity among very low- and trace-positive participants.

**Table 2:** Sensitivity and specificity of prior TB history with regards to culture positivity for the full dataset, training set and test set.

	Training Dataset			Testing Dataset			Full Dataset		
	Culture Negative (n = 56)	Culture Positive (n = 11)	*P*-value	Culture Negative (n = 17)	Culture Positive (n = 6)	*P*-value	Culture Negative (n = 73)	Culture Positive (n = 17)	*P*-value

Prior TB History—n(%)									
No	33 (58.9)	6 (54.5)		9 (52.9)	5 (83.3)		42 (57.5)	11 (64.7)	
Yes	23 (41.1)	5 (45.5)	1.00	8 (47.1)	1 (16.7)	0.34	31 (42.5)	6 (35.3)	0.79
Specificity (%)	33/56 (58.9)			9/17 (52.9)			42/73 (57.5)		
Sensitivity (%)	5/11 (45.5)			1/6 (16.7)			6/17 (35.3)		

Data represents no (%). Comparisons were made using Fisher’s exact test.

## Data Availability

All code used to generate these results are available on GitHub at: https://github.com/lopkattya/TRACE4_analysis.git. The TRACE4 signature has been added to the TBSP R package as Lopez_TRACE_4 (according to their nomenclature).
