# Supplementary material for: Development of a four-gene host signature for paucibacillary TB among symptomatic individuals with sputum Xpert MTB/RIF Ultra very low and trace results
Source: Int J Infect Dis. Author manuscript; Available in PMC 2026 Oct 26. (PMC13698042; doi:10.1016/j.ijid.2026.108763)
Supplement: Supplementary file 1 [file NIHMS2212165-supplement-Supplementary_material.docx]

**Table S1**. Baseline characteristics.

| Characteristic | Mtb Culture Positive  n = 17 | Mtb Culture Negative  n = 73 | | p-value |
| --- | --- | --- | --- | --- |
| Xpert MTB/RIF Ultra – no. (%) |  |  | | **<0.001***** |
| Trace | 3 (17.6) | 52 (71.2) | |  |
| Very low | 14 (82.4) | 21 (28.8) | |  |
| ^^^Age, median (IQR), years | 32 (26-38) | 38 (30-45) | | 0.17 |
| Sex - no. (%) |  |  | | 0.28 |
| Male | 7 (41.2) | 42 (57.5) | |  |
| Female | 10 (58.8) | 31 (42.5) |  | |
| Site - no. (%) |  |  | 0.39 | |
| Kenya | 7 (41.2) | 20 (27.4) |  | |
| South Africa | 6 (35.3) | 24 (32.9) |  | |
| Uganda | 4 (23.5) | 29 (39.7) |  | |
| HIV-positive - no. (%) |  |  | 0.42 | |
| Yes | 11 (64.7) | 37 (50.7) |  | |
| No | 6 (35.3) | 36 (49.3) |  | |
| Prior TB - no. (%) |  |  | 0.79 | |
| Yes | 6 (35.3) | 31 (42.5) |  | |
| No | 11 (64.7) | 42 (57.5) |  | |
| Symptoms - no. (%) |  |  |  | |
| Cough | 17 (100.0) | 73 (100.0) | 1.00 | |
| Fever | 13 (76.5) | 50 (68.5) | 0.77 | |
| Night sweats | 8 (47.1) | 44 (60.3) | 0.42 | |
| Weight loss | 13 (76.5) | 46 (68.5) | 0.40 | |
| Hemoptysis | 2 (11.8) | 10 (13.7) | 1 | |
| Smear microscopy - no. (%) |  |  | 0.34 | |
| Negative | 16 (94.1) | 72 (98.6) |  | |
| 1+ | 1 (5.9) | 0 (0.0) |  | |
| 3+ | 0 (0.0) | 1 (1.4) |  | |
| ^◊^ Diagnosis at day 7 follow-up - no. (%) |  |  | **<0.005**** | |
| Not diagnosed with TB | 4 (25.0) | 46 (65.7) |  | |
| Active pulmonary TB | 12 (75.0) | 23 (32.9) |  | |
| Pulmonary and extrapulmonary TB | 0 (0.0) | 1 (1.4) |  | |

Data represents no (%) or median and interquartile range (IQR). Comparisons were made using the Fisher’s exact test or Mann-Whitney U test^^^.

^◊^ Four individuals had missing values for TB diagnosis: one participant in the culture-positive group and three participants in the culture-negative group. Percentages for day 7 TB diagnosis status were calculated among participants with available day 7 data.

**p < 0.01, *** p < 0.001.

**Table S2.** Baseline characteristics in the training set.

| **Characteristic** | **Mtb Culture Positive**  **n = 11** | **Mtb Culture Negative**  **n = 56** | **p-value** |
| --- | --- | --- | --- |
| **Xpert MTB/RIF Ultra – no. (%)** |  |  | 0.02 |
| Trace | 3 (27.0) | 37 (66.0) |  |
| Very low | 8 (73.0) | 19 (34.0) |  |
| **^^^**Age, median (IQR), years | 32 (28-39) | 39.5 (32.75 – 46.25) | 0.256 |
| **Sex - no. (%)** |  |  | 0.33 |
| Male | 4 (36.0) | 31 (55.0) |  |
| Female | 7 (64.0) | 25 (45.0) |  |
| **Site - no. (%)** |  |  | 0.32 |
| Kenya | 5 (45.0) | 13 (23.0) |  |
| South Africa | 4 (36.0) | 24 (43.0) |  |
| Uganda | 2 (18.0) | 19 (34.0) |  |
| **HIV-positive - no. (%)** |  |  | 1.00 |
| Yes | 6 (45.0) | 30 (54.0) |  |
| No | 5 (55.0) | 26 (36.0) |  |
| **Prior TB - no. (%)** |  |  | 1.00 |
| Yes | 5 (45.0) | 23 (41.0) |  |
| No | 23 (55.0) | 33 (59.0) |  |
| **Symptoms - no. (%)** |  |  |  |
| Cough | 11 (100.0) | 56 (100.0) | - |
| Fever | 9 (82.0) | 39 (70.0) | 0.72 |
| Night sweats | 6 (55.0) | 34 (61.0) | 0.75 |
| Weight loss | 7 (64.0) | 33 (59.0) | 1.00 |
| Hemoptysis | 1 (9.0) | 8 (14.0) | 1.00 |
| **Smear microscopy - no. (%)** |  |  | 0.30 |
| Negative | 10 (91.0) | 55 (98.0) |  |
| 1+ | 1 (9.0) | 0 (0.0) |  |
| 3+ | 0 (0.0) | 1 (2.0) |  |
| ^◊^ **Diagnosis at day 7 follow-up -  no. (%)** |  |  | 0.29 |
| Not diagnosed with TB | 4 (40.0) | 35 (66.0) |  |
| Active pulmonary TB | 6 (60.0) | 17 (32.0) |  |
| Pulmonary and extrapulmonary TB | 0 | 1 (2.0) |  |

Data represents no (%) or median and interquartile range (IQR). Comparisons were made using the Fisher’s exact test or Mann-Whitney U test^^^.

^◊^ Four individuals had missing values for TB diagnosis: one participant in the culture-positive group and three participants in the culture-negative group. Percentages for day 7 TB diagnosis status were calculated among participants with available day 7 data.

**p < 0.01, *** p < 0.001

**Table S3**. Baseline characteristics in the test set.

| **Characteristic** | **Mtb Culture Positive**  **n = 6** | **Mtb Culture Negative**  **n = 17** | **p-value** |
| --- | --- | --- | --- |
| **Xpert MTB/RIF Ultra – no. (%)** |  |  |  |
| Trace | 0 (0.0) | 15 (88.0) | **<0.001** |
| Very low | 6 (100.0) | 2 (12.0) |  |
| **^^^**Age, median (IQR), years | 30 (24.5-36.5) | 32 (28-41) | 0.369 |
| **Sex - no. (%)** |  |  |  |
| Male | 3 (50.0) | 11 (65.0) |  |
| Female | 3 (50.0) | 6 (35.0) | 0.64 |
| **Site - no. (%)** |  |  |  |
| Kenya | 2 (33.3) | 7 (41.0) | 0.10 |
| South Africa | 2 (33.3) | 0 (0.0) |  |
| Uganda | 2 (33.3) | 10 (59.0) |  |
| **HIV-positive - no. (%)** |  |  |  |
| Yes | 5 (83.0) | 7 (41.0) | 0.155 |
| No | 1 (17.0) | 10 (59.0) |  |
| **Prior TB - no. (%)** |  |  |  |
| Yes | 1 (17.0) | 8 (47.0) | 0.34 |
| No | 5 (83.0) | 9 (53.0) |  |
| **Symptoms - no. (%)** |  |  |  |
| Cough | 6 (100.0) | 17 (100.0) | - |
| Fever | 4 (68.0) | 11 (65.0) | 1.00 |
| Night sweats | 2 (33.3) | 10 (59.0) | 0.37 |
| Weight loss | 6 (100.0) | 13 (76.0) | 0.54 |
| Hemoptysis | 1 (17.0) | 2 (12.0) | 1.00 |
| **Smear microscopy - no. (%)** |  |  |  |
| Negative | 6 (100.0) | 17 (100.0) | - |
| **Diagnosis at day 7 follow-up -  no. (%)** |  |  |  |
| Not diagnosed with TB | 0 (0.0) | 11 (0.65) | **0.01** |
| Active pulmonary TB | 6 (100.0) | 6 (0.35) |  |

Data represents no (%) or median and interquartile range (IQR). Comparisons were made using the Fisher’s exact test or Mann-Whitney U test^^^.

**p < 0.01, *** p < 0.001

**Table S4**. Evaluation of the performance of thirteen published TB signatures.

| Signature | Area Under Curve (AUC) values | p-value |
| --- | --- | --- |
| Maertzdorf4 | 0.73 | 0.005** |
| Zak-RISK16 | 0.70 | 0.02* |
| Darboe-RISK11 | 0.70 | 0.02* |
| Roe3 | 0.63 | 0.12 |
| Zhao-NANO6 | 0.62 | 0.08 |
| PennNich-RISK6 | 0.59 | 0.25 |
| Suliman-RISK4 | 0.58 | 0.23 |
| Leong-RISK29 | 0.57 | 0.38 |
| Sloot-HIV2 | 0.56 | 0.39 |
| Jacobsen3 | 0.53 | 0.67 |
| Kulkarni-HIV2 | 0.52 | 0.82 |
| Sweeney-OD3 | 0.51 | 0.95 |
| Sambarey-HIV10 | 0.51 | 0.89 |

*p < 0.05, **p < 0.01. Comparison between culture-positive and culture-negative among individuals with Ultra very low or trace results.

**Figure S1.** Gene importance and signature derivation. 1). Four examples of variable importance gene scores generated by H2O platform, 2). Boxplot comparison of AUC performance for candidate TB signatures using the TBSignatureProfiler (TBSP) tool. The asterisk (*) corresponds to our four-gene host signature. GBM: Gradient Boosting Machine, GLM: Generalized Linear Model, MLP: Multilayer Perception, XGBOOST: eXtreme Gradient Boosting.

**Table S5.** Signature score performance metrics and cutoff derivation for classifying culture status in Ultra very low or trace subjects from the training dataset (n = 67).

|  | **Culture Negative vs Culture Positive among Trace Subjects**  **Training Dataset (n = 67)** | | | | | | |
| --- | --- | --- | --- | --- | --- | --- | --- |
| **Signature** | **Diagnostic Sensitivity**  **[95% CI]^1^** | **Diagnostic Specificity**  **[95% CI]^2^** | **Triage Sensitivity [95% CI]^3^** | **Triage Specificity [95% CI]^4^** | **AUC**  **[95% CI]** | **Maximum Youden’s Index**  **(J statistic)** | **Signature Score Optimal Cutoff** |
| TRACE4 | 0.36 [0.09, 0.73] | 0.82 [0.61, 0.95] | 0.91 [0.73, 1.00] | 0.80 [0.54, 0.91] | 0.89 [0.80, 0.96] | 0.71 | 0.29 |
| Zak-RISK16 | 0.00 [0.00, 0.36] | 0.46 [0.14, 0.79] | 0.54 [0.18, 0.82] | 0.25 [0.11, 0.66] | 0.66 [0.48, 0.83] | 0.29 | 0.73 |
| Darboe-RISK11 | 0.00 [0.00, 0.36] | 0.41 [0.18, 0.75] | 0.45 [0.18, 0.82] | 0.36 [0.13, 0.66] | 0.67 [0.49, 0.83] | 0.37 | 0.73 |
| Maertzdorf4 | 0.00 [0.00, 0.45] | 0.66 [0.05, 0.84] | 0.73 [0.27, 1.00] | 0.66 [0.04, 0.80] | 0.75 [0.56, 0.88] | 0.57 | 0.19 |
| Zhao-NANO6 | 0.00 [0.00, 0.00] | 0.36 [0.23, 0.77] | 0.54 [0.18, 0.82] | 0.36 [0.21, 0.64] | 0.64 [0.49, 0.79] | 0.30 | 0.17 |
| Roe3 | 0.09 [0.00, 0.45] | 0.38 [0.20, 0.71] | 0.45 [0.18, 0.82] | 0.34 [0.16, 0.54] | 0.65 [0.47, 0.82] | 0.26 | 0.50 |
| Leong-RISK29 | 0.00 [0.00, 0.00] | 0.50 [0.16, 0.66] | 0.45 [0.00, 0.73] | 0.27 [0.13, 0.61] | 0.58 [0.43, 0.73] | 0.32 | -0.04 |
| PennNich-RISK6 | 0.00 [0.00, 0.27] | 0.18 [0.07, 0.61] | 0.27 [0.00, 0.55] | 0.14 [0.05, 0.57] | 0.53 [0.34, 0.70] | 0.21 | -0.06 |
| Suliman-RISK4 | 0.00 [0.00, 0.00] | 0.34 [0.13, 0.61] | 0.45 [0.00, 0.73] | 0.29 [0.09, 0.52] | 0.56 [0.39, 0.73] | 0.20 | 0.31 |
| Sweeney-OD3 | 0.00 [0.00, 0.00] | 0.39 [0.00, 0.66] | 0.45 [0.00, 0.73] | 0.23 [0.00, 0.54] | 0.55 [0.37, 0.73] | 0.21 | -0.21 |
| Kulkarni-HIV2 | 0.09 [0.00, 0.27] | 0.29 [0.05, 0.50] | 0.18 [0.00, 0.55] | 0.16 [0.04, 0.45] | 0.49 [0.31, 0.67] | 0.10 | -0.27 |
| Sloot-HIV2 | 0.00 [0.00, 0.09] | 0.27 [0.05, 0.48] | 0.27 [0.00, 0.64] | 0.18 [0.04, 0.41] | 0.48 [0.30, 0.65] | 0.09 | -0.52 |
| Sambarey-HIV10 | 0.00 [0.00, 0.00] | 0.34 [0.20, 0.66] | 0.45 [0.00, 0.73] | 0.30 [0.18, 0.55] | 0.56 [0.42, 0.71] | 0.27 | -0.20 |
| Jacobsen3 | 0.09 [0.00, 0.36] | 0.34 [0.00, 0.55] | 0.18 [0.00, 0.45] | 0.27 [0.00, 0.46] | 0.51 [0.33, 0.69] | 0.18 | -0.26 |

^1^Calculated at 0.98 specificity, the WHO TPP required minimum for a biomarker-based non-sputum-based diagnostic assay for detecting TB.

^2^Calculated at 0.75 sensitivity, the WHO TPP required minimum for a biomarker-based non-sputum-based diagnostic assay for detecting pulmonary TB in adults.

^3^Calculated at 0.70 specificity, the WHO TPP required minimum for a biomarker-based non-sputum-based screening assay for detecting TB.

^4^Calculated at 0.90 sensitivity, the WHO TPP required minimum for a biomarker-based non-sputum-based screening assay for detecting TB.

**Table S6.** Signature score performance metrics and cutoff derivation for classifying culture status in Ultra very low or trace subjects from the test dataset (n = 23).

|  | **Culture Negative vs Culture Positive among Trace Subjects - Test Dataset (n = 23)** | | | | | | | |
| --- | --- | --- | --- | --- | --- | --- | --- | --- |
| **Signature** | **Diagnostic Sensitivity [95% CI]^1^** | **Diagnostic Specificity [95% CI]^2^** | **Triage Sensitivity [95% CI]^3^** | **Triage Specificity [95% CI]^4^** | **AUC [95% CI]** | **Sensitivity at Derived Cutoff^5^** | **Specificity at Derived Cutoff^5^** | **Signature Score Derived Cutoff^5^** |
| **TRACE4** | **0.33 [0.00, 0.83]** | **0.82 [0.59, 1.00]** | **1.00 [0.33, 1.00]** | **0.76 [0.59, 0.94]** | **0.88 [0.71, 0.99]** | **0.83** | **0.82** | **0.2867597** |
| Zak-RISK16 | 0.17 [0.00, 0.67] | 0.65 [0.12, 0.94] | 0.67 [0.17, 1.00] | 0.29 [0.12, 0.82] | 0.74 [0.49, 0.93] | 0.50 | 0.88 | 0.7278184 |
| Darboe-RISK11 | 0.33 [0.00, 0.83] | 0.65 [0.12, 1.00] | 0.67 [0.17, 1.00] | 0.29 [0.12, 0.88] | 0.76 [0.51, 0.96] | 0.17 | 1.00 | 0.7338710 |
| Maertzdorf4 | 0.00 [0.00, 0.50] | 0.47 [0.24, 0.88] | 0.67 [0.00, 1.00] | 0.47 [0.24, 0.82] | 0.70 [0.47, 0.90] | 0.67 | 0.76 | 0.1892589 |
| Zhao-NANO6 | 0.17 [0.00, 0.67] | 0.18 [0.00, 0.88] | 0.50 [0.00, 1.00] | 0.06 [0.00, 0.65] | 0.58 [0.25, 0.87] | 0.50 | 0.65 | 0.1739385 |
| Roe3 | 0.00 [0.00, 0.50] | 0.47 [0.06, 0.88] | 0.50 [0.00, 1.00] | 0.18 [0.00, 0.71] | 0.63 [0.36, 0.86] | 0.00 | 0.88 | 0.4990315 |
| Leong-RISK29 | 0.00 [0.00, 0.00] | 0.24 [0.00, 0.82] | 0.33 [0.00, 1.00] | 0.12 [0.00, 0.71] | 0.53 [0.26, 0.79] | 0.67 | 0.18 | -0.0423499 |
| PennNich-RISK6 | 0.00 [0.00, 0.17] | 0.47 [0.24, 0.82] | 0.50 [0.00, 1.00] | 0.41 [0.18, 0.71] | 0.62 [0.39, 0.83] | 0.67 | 0.53 | -0.0641207 |
| Suliman-RISK4 | 0.00 [0.00, 0.00] | 0.18 [0.00, 0.41] | 0.00 [0.00, 0.50] | 0.00 [0.00, 0.35] | 0.27 [0.08, 0.53] | 0.33 | 0.76 | 0.3059190 |
| Sweeney-OD3 | 0.17 [0.00, 0.50] | 0.24 [0.00, 0.76] | 0.33 [0.00, 0.83] | 0.12 [0.00, 0.65] | 0.53 [0.25, 0.80] | 0.67 | 0.29 | -0.2146770 |
| Kulkarni-HIV2 | 0.00 [0.00, 0.33] | 0.47 [0.00, 0.88] | 0.50 [0.00, 1.00] | 0.12 [0.00, 0.71] | 0.59 [0.33, 0.84] | 0.67 | 0.29 | -0.2719085 |
| Sloot-HIV2 | 0.00 [0.00, 0.00] | 0.24 [0.06, 0.53] | 0.17 [0.00, 0.50] | 0.24 [0.06, 0.47] | 0.38 [0.16, 0.62] | 1.00 | 0.12 | -0.5202819 |
| Sambarey-HIV10 | 0.00 [0.00, 0.00] | 0.29 [0.00, 0.59] | 0.17 [0.00, 0.50] | 0.06 [0.00, 0.47] | 0.39 [0.16, 0.66] | 0.33 | 0.71 | -0.2045298 |
| Jacobsen3 | 0.00 [0.00, 0.00] | 0.29 [0.06, 0.53] | 0.17 [0.00, 0.50] | 0.18 [0.06, 0.47] | 0.41 [0.18, 0.65] | 0.17 | 0.71 | -0.2635175 |

^1^Calculated at 0.98 specificity, the WHO TPP required minimum for a biomarker-based non-sputum-based diagnostic assay for detecting TB.

^2^Calculated at 0.75 sensitivity, the WHO TPP required minimum for a biomarker-based non-sputum-based diagnostic assay for detecting pulmonary TB in adults.

^3^Calculated at 0.70 specificity, the WHO TPP required minimum for a biomarker-based non-sputum-based screening assay for detecting TB.

^4^Calculated at 0.90 sensitivity, the WHO TPP required minimum for a biomarker-based non-sputum-based screening assay for detecting TB.

**Table S7**. Performance metrics of prior TB history, clinical TB diagnosis by day 7, and the top four transcriptomic signatures for classifying culture status among Ultra “very low” or “trace” Xpert-positive participants based on WHO ***triage*** test benchmarks (test set n = 23, full dataset n = 67).

|  | **Specificity, Test Set**  **[95% CI]** | **Sensitivity,**  **Test Set**  **[95% CI]** | **Specificity,**  **Full Dataset**  **[95% CI]** | **Sensitivity,**  **Full Dataset**  **[95% CI]** |
| --- | --- | --- | --- | --- |
| Prior TB history^2^ | 0.53 [0.29, 0.76] | 0.17 [0.01, 0.64] | 0.58 [0.45, 0.69] | 0.35 [0.15, 0.61] |
| Clinical TB diagnosis by day 7 | 0.65 [0.39, 0.85] | 1.00 [0.52, 1.00] | 0.66 [0.53, 0.76] | 0.75 [0.47, 0.92] |
|  | | | | |
|  | **Specificity, Test Set [95% CI]^1^** | **Sensitivity, Test Set**  **[95% CI]** | **HRS cutoff** | **AUC [95% CI]** |
| **TRACE4** | 0.76 [0.59, 0.94] | 0.90 | 0.052 | 0.88 [0.71, 0.99] |
| Darboe-RISK11 | 0.29 [0.12, 0.88] | 0.90 | -0.629 | 0.76 [0.51, 0.96] |
| Zak-RISK16 | 0.29 [0.12, 0.82] | 0.90 | -0.695 | 0.74 [0.49, 0.93] |
| Maertzdorf4 | 0.47 [0.24, 0.82] | 0.90 | -0.302 | 0.70 [0.47, 0.90] |

^1^Specificity is calculated at 0.90 sensitivity, the WHO TPP required minimum for a biomarker-based non-sputum-based TB screening (triage) assay.

^2^Prior TB history: consideration of a trace Ultra result as likely remnant Mtb DNA from prior TB, and thus TB-negative status. One individual is missing results for prior TB history in the test set, and four individuals are missing results in the full dataset.

Diagnostic performance was assessed based on association with culture positivity among very low- and trace-positive participants.

**Table S8**. Datasets for TRACE4 signature validation.

Abbreviations: GSVA; gene set variation analysis, LTBI; latent tuberculosis infection, PTB; pulmonary tuberculosis, ssGSEA; single-sample gene set enrichment analysis, OD; Other respiratory diseases, PLAGE; pathway level analysis of gene expression, TB; tuberculosis.

| Gene Expression  Dataset  (Reference ID) | Country | Comparison | Scoring Method and area under the curve (AUC) |
| --- | --- | --- | --- |
| GSE19442 | South Africa | PTB vs LTBI | GSVA = 0.87  ssGSEA = 0.64  PLAGE = 0.98 |
| GSE69581 | South Africa | PTB vs LTBI | GSVA = 0.87  ssGSEA = 0.64  PLAGE = 0.98 |
| GSE73408 | United States | PTB vs LTBI    PTB vs OD | GSVA = 0.83  ssGSEA = 0.91  PLAGE = 0.84  GSVA = 0.76  ssGSEA = 0.76 |
| GSE101705 | India | PTB vs LTBI | GSVA = 0.88  ssGSEA = 0.92  PLAGE = 0.90 |
